# Decreased Cognitive/CNS Function in Young Adults at Risk for Hypertension: Effects of Sleep Deprivation

**DOI:** 10.1155/2012/989345

**Published:** 2012-01-24

**Authors:** James A. McCubbin, Hannah Peach, DeWayne D. Moore, June J. Pilcher

**Affiliations:** Department of Psychology, Clemson University, Clemson, SC 29634, USA

## Abstract

Hypertension has been linked to impaired cognitive/CNS function, and some of these changes may precede development of frank essential hypertension. The stress and fatigue of sleep deprivation may exacerbate these cognitive changes in young adults at risk. We hypothesize that individuals at risk for hypertension will show significant declines in cognitive function during a night of sleep deprivation. Fifty-one young adults were recruited for 28-hour total sleep deprivation studies. Hypertension risk was assessed by mildly elevated resting blood pressure and by family history of hypertension. A series of cognitive memory tasks was given at four test sessions across the sleep deprivation period. Although initially comparable in cognitive performance, persons at risk showed larger declines across the night for several indices of working memory, including code substitution, category, and order recall. These results suggest that cognitive/CNS changes may parallel or precede blood pressure dysregulation in the early stages of hypertension development. The role of CNS changes in the etiology of essential hypertension is discussed.

## 1. Introduction

Hypertensive patients have impaired cognitive and CNS function, and some of these changes may precede development of frank essential hypertension. Subtle cognitive changes in younger persons at risk for hypertension may become more readily apparent during the systemic stress of sleep deprivation. We hypothesize that young adults at risk for hypertension will show significant declines in cognitive function during a night of sleep deprivation.

Cognitive decline in older persons with advanced hypertension is especially well documented and likely represents, at least in part, the damaging effects of sustained high blood pressure on CNS microvasculature, and, hence, brain function [1–5]. For example, hypertension is associated with increased likelihood of dementia in older adults [6–9]. Some of the cognitive decline in older hypertensive patients can be reversed by pharmacological, dietary, and weight loss approaches to blood pressure reduction [10], indicating that chronic high blood pressure can have a damaging effect, either directly or indirectly, on brain function.

However, some cognitive changes may precede the development of frank clinical hypertension, suggesting a more complex association between the CNS and blood pressure in the development of essential hypertension. For example, decreased cognitive/CNS function has been recently found in middle aged and younger hypertensives [11, 12], young people with mildly elevated blood pressure [13], and in normotensives with a positive family history of hypertension [14, 15]. These findings suggest that relatively minor preclinical changes in blood pressure are associated with subtle changes in brain function. Therefore, CNS changes may parallel, precede, and/or contribute to blood pressure elevations, especially in young persons whose cerebral vasculature has not been exposed to the deleterious effects of significant and sustained blood pressure elevations. Thus, the study of cognitive changes in early stages of hypertension development may provide insight into the possible neurogenic precursors and/or etiologic mechanisms of essential hypertension. Interestingly, the systemic stress and fatigue of sleep deprivation may exacerbate both cognitive and circulatory changes in young adults at risk for development of hypertension. For example, sleep deprivation has been shown to disrupt executive attention, working memory and other higher cognitive functions [16]. Moreover, neural systems that underlie executive function are especially vulnerable to the effects of sleep deprivation in some individuals [17]. Young adults at risk for hypertension development later in life show a spectrum of neural, endocrine and circulatory changes during stress [18–20], possibly including the systemic stress of sleep deprivation. For example, some of studies from our sleep laboratory recently showed that a night of sleep deprivation increased blood pressure in young adults with a positive family history of hypertension versus negative family history controls [21]. The present study, part of that larger series of sleep deprivation studies, focuses on the effect of sleep deprivation on cognitive function in persons at risk for hypertension. We hypothesize that persons at risk for hypertension will show declines in higher cognitive performance during sleep deprivation.

## 2. Materials and Methods

### 2.1. Participants

Participants were fifty-one volunteers (28 males and 23 females) with an average age of 22.9 years. Young adult study participants were recruited from campus and the surrounding community with ages ranging from 19–32 years old. This sample included graduate and undergraduate students, university employees, and community citizens. Average body weight was 148 ± 25.7 lbs., and average body mass index was 22.6 ± 3.26. The participants completed questionnaires about personal and family medical history, sleep habits, and alcohol and tobacco use. Only persons reporting a regular diurnal sleep/wake cycle were selected for participation. The final study population was a healthy, normal sample without sleep disorders or significant cardiovascular, neurological, endocrine, or psychiatric disease. All subjects refrained from use of caffeine and tobacco and exercise throughout the study period. Informed consent was obtained from each subject before participation. All procedures were approved by the Clemson University Institutional Review Board.

### 2.2. Procedures

The present investigation is part of a larger series of studies of sleep deprivation and sustained operations. Procedures have been described in detail by Pilcher and coworkers [22]. Participants were recruited through posted flyers detailing the two-day sleep deprivation study. After screening, volunteers met with researchers three days before the study to discuss the consent form, study procedures, and instructions in completing the study. The participants were instructed to sleep for eight hours each night for the three days prior to the sleep deprivation study. Participants were also instructed not to drink alcohol the day before the study and were told not to consume any caffeine or substances high in sugar (e.g., candy bar) the morning of the study. Participants were given an Actiwatch (Mini Mitter Company Inc., Bend, OR, USA) that they wore for the three days prior to the study. Actiwatches were worn on the nondominant arm to record wrist movement indices of normal sleep/wake patterns.

Participants also received a sleep log to provide information on sleep habits prior to the onset of the study. These sleep logs were to be completed each morning of the three days prior to the study. The sleep logs included questions inquiring about sleep quality, time going to bed, time getting out of bed, and napping throughout the day. Analysis of Actiwatch data confirmed accuracy of sleep logs to verify adherence to instructions. Participants reported at 9:30 AM on Day 1 and were transported to the residential sleep laboratory. Food and noncaffeinated beverages were provided throughout the study.

The participants completed a series of tasks and questionnaires and were given scheduled breaks and meals throughout the study period. Training on the cognitive tasks was completed in two periods on Day 1. Four testing sessions were scheduled at approximately 8:30 PM, 1:00 AM, 5:30 AM, and 10:00 AM across the sleep deprivation period. The study ended on Day 2 when participants were transported back to their residences and instructed to sleep before operating heavy equipment or driving. 

### 2.3. Measures

#### 2.3.1. Sleep and Health

Questionnaires were administered to obtain information on typical sleep patterns and individual and familial medical history for each participant. The Pittsburgh Sleep Quality Index (PSQI) was used to assess recent sleep quality. Its reliability and validity has been verified by Buysse and coworkers [23].

#### 2.3.2. Blood Pressure

Resting blood pressure (BP) was measured upon arrival and departure at approximately 11 PM each day and at approximately 8:30 PM, 1:00 AM, 5:30 AM, and 10:00 AM over the study period. Electronic blood pressure measurements were taken using GE Dinamap Pro100 machines (Medical Solutions, Minneapolis, MN.). Dinamap performance was verified on a regular basis for zero offset, integral offset, and gain using a mercury manometer. All devices performed within manufacturer tolerances. Research assistants were trained on theory and application of blood pressure determination using both auscultatory and oscillometric techniques, including use of appropriate cuff sizes and other American Heart Association guidelines for blood pressure determination [24]. At each blood pressure determination, participants sat quietly in a comfortable armchair for five minutes prior to taking five BP readings at one-minute intervals. The last three readings were averaged to create a single, stable resting BP index at each time period.

#### 2.3.3. Memory Tasks

The participants also completed five subtests from the Automated Neuropsychological Assessment Metrics (ANAM). This measure is a battery of cognitive tests developed by the Office of Military Performance Assessment (OMPAT; Washington, DC). The ANAM tests have strong correlations with traditional measures of neuropsychological functioning, high test-retest reliability (typically = 0.80, 0.95 range), high differential stability, and a large database of studies providing construct validity [25]. The measure includes five different tasks, all of which were used for the purposes of this study. The tasks were given in the following order for each administration (testing and training): Code Substitution Learning (CDS), Code Substitution Immediate Memory Recall (CDI), Sternberg Memory Recall (ST6), Continuous Performance Test (CPT), and Code Substitution Delayed Memory Recall (CDD). Participants were given the battery 5 times during the training sessions to control for learning effects and then once during each of the 4 testing sessions. ANAM tasks took approximately 12–14 minutes to complete. Apart from the fixed order required by ANAM components, task order was fixed within subjects, but counterbalance between subjects.

#### 2.3.4. Code Substitution Learning (CDS)

Participants were presented with a set of symbols (*δ*, ¥, æ, *◄*, *╣*, *↕*, Ω, *√*, *≡*) that corresponded to a number, 1–9. With the key on the screen for reference, they were given a symbol and number and asked to respond if the pair correctly matched the given key. Participants were given 72 trials during each administration of the task. Each stimulus was displayed for 4000 milliseconds, and the time allowed for response was 4200 milliseconds before the next stimulus was displayed.

#### 2.3.5. Code Substitution Immediate Recall (CDI)

Participants were asked to recall the previous key of numbers and symbols that was given during the Code Substitution Practice task. They were presented with a symbol and number pair and indicated if the stimulus matched the previously memorized key. Participants were given 18 trials during each administration of the task. Each stimulus was displayed for 4000 milliseconds, and the time allowed for response was 4200 milliseconds before the next stimulus was displayed.

#### 2.3.6. Code Substitution Delayed Recall (CDD)

After completing two additional tasks (see ST6 and CPT below), participants were asked to recall the memorized key given during the Code Substitution Practice task after approximately 10 minutes had elapsed since it was presented. The participants were given 36 trials in the CDD. Each stimulus was displayed for 4000 milliseconds, and the time allowed for response was 4200 milliseconds before the next stimulus was displayed.

#### 2.3.7. Sternberg Memory Recall (ST6)

Participants were given a set of 6 letters to memorize. They could review the letters until pressing the space bar to continue the task. Single “probe” letters were then flashed on the screen. The participants then indicated if the stimulus was part of the memory set. The probe was displayed on the screen for 1400 ms. The maximum time to respond to each presented stimulus was 1500 milliseconds.

#### 2.3.8. Category Recall and Order Recall

Participants completed a Category Recall (CatRecall) task presented on a computer to test memory. The CatRecall memory task was developed using E-prime at the University of Maryland's Center for Advanced Study of Language and derived from previously formed category memory tasks [19]. At the beginning of the task, the participant was presented with 6 different categories containing 8 examples within each category, for example, animal: cat, bird, dog, horse, lion, mouse, snake and wolf. Subjects were allowed to study the words and categories for an unlimited amount of time before continuing.

Participants were presented with a “memory list,” which was a series of six words, one from each category. Each word in the memory list was displayed on the computer screen for 400 milliseconds. Participants were then presented with a probe category such as “music” from the 6 available category names. The probe category was displayed for 3000 ms. Participants were then asked to identify which word from the memory list was originally included in the category presented.

The order recall task presented participants with a memory list of 6 words, one at a time. Participants were asked to remember the 6 words in order, and when presented with a cue word from the memory list, to correctly choose the word from the memory list that followed the cue word.

#### 2.3.9. Continuous Performance Test (CPT)

Participants were presented single-digit numbers (1–9) one at a time. Once a second number appeared, they indicated whether that number was the same as the previously presented number (a 1-back task). Participants were presented with 179 numbers resulting in 178 trials. This task was divided into two sections: with feedback (1 minute) and without feedback (4 minutes). The probe was displayed on the screen for 1000 ms, and participants were given a maximum of 1500 milliseconds to respond.

#### 2.3.10. Classifications of Subjects by Risk for Hypertension

Risk for subsequent development of hypertension was determined in two different ways, by resting blood pressure levels and by reported parental history of hypertension. Classification of risk by resting blood pressure levels was based on criteria outlined in the Seventh Report of the Joint National Committee on the Prevention, Detection, Evaluation, and Treatment of High Blood Pressure (JNC-7) [26]. Briefly, subjects rested in a seated position while blood pressure determinations were made on two different days at approximately the same time each day. Classifications were based on the average of two readings each day. Participants were classified as normal if they had no blood pressure above 120 mmHg systolic and 80 mmHg diastolic on both days of measurement. Participants were classified as prehypertensive if they had blood pressure between 120–139 systolic or 80–89 diastolic on both days of measurement. No participants were classified above the prehypertensive range. Risk associated with moderately elevated pressures in young adults has been shown by several studies. For example, the level of pressure in young adults has been shown to predict both the level of pressure and incidence of essential hypertension in later life [27, 28]. Moreover, a recent meta-analysis showed that persons with SBP 130–139 or DBP 85–89 have up to 55% increased risk of stroke [29].

Participants were classified with a positive family history of hypertension if one or both biological parents were identified as having been diagnosed with essential hypertension by a physician. Participants with no reported parental hypertension were classified as negative family history. Validity of self-reported parental hypertension has been consistently demonstrated through direct contact with parents and parents' physicians [30–32].

### 2.4. Data Analyses

All BP data were entered into Microsoft Excel and imported into SPSS (IBM, Armonk, NY, USA) for statistical analyses. Cognitive performance measures were initially analyzed in a 2 × 2 × 4 (Risk × Sex × Time) design using the SPSS General Linear Model with Time as the within subjects variable, Risk and Sex as between subjects variables, and multivariate *F* tests for main effects and interactions with Time. One set of Risk analyses were conducted using JNC-7 grouping of blood pressure (prehypertensive versus normotensive). Additional analyses were conducted using family history of hypertension (positive versus negative family history) as an alternate Risk variable. Post hoc simple main effects were assessed using Fisher's LSD.

## 3. Results

Means and standard errors for blood pressures and cognitive performance of all participants at the four test sessions are shown in Table 1. Repeated measures ANOVAs on cognitive performance generally showed significant sleep deprivation-induced declines in performance for all subjects across testing periods. For example, when collapsed across risk status, significant declines across time were observed for code substitution simultaneous (*F*(3, 41) = 7.71, *P* < .001), code substitution immediate (*F*(3, 41) = 8.903, *P* < .001), code substitution delayed (*F*(3, 41) = 21.876, *P* < .001), and continuous performance (*F*(3, 41) = 11.955, *P* < .001).

### 3.1. Effects of Sleep Deprivation on Blood Pressures in High- and Low-Risk Groups

Using the JNC-7 criteria for classification of blood pressure, 19.6% of participants (10 of 51) were classified as prehypertensive. Average age was 21.9 years for the prehypertensive group and 23.0 years for the normal group. Using family history of hypertension as an index of risk, 23.5% of participants (12 of 51) reported a positive family history of essential hypertension in at least one biological parent. Average age was 23.0 years for the positive family history group and 22.7 years for the negative family history group. There were no significant group differences in age, weight, body mass index, or distribution of sex among risk groups (*P* > .05). Only 3.9% of participants (2 of 51) had both a prehypertensive classification and a positive family history of hypertension.

Results for blood pressures across time and by risk groups have been reported elsewhere [16]. Briefly, the prehypertensive groups showed significantly higher resting systolic (multivariate *F*(1, 46) = 20.839, *P* < .001, *η* ^2^ = .312) and diastolic [multivariate *F*(1, 48) = 4.638, *P* = .036, *η* ^2^ = .088] blood pressure across all time periods. Using family history of hypertension for risk categorization, there were no significant initial baseline differences in blood pressure between high- and low-risk groups, however the Family History X Time interaction for diastolic blood pressure was significant [multivariate *F*(3, 46) = 4.574, *P* = .007, *η* ^2^ = .230], indicating that diastolic blood pressure for the two family history groups significantly diverged across the night of sleep deprivation, with slight decreases across the night in subjects with negative family history and concomitant increases in subjects with positive family history of hypertension.

### 3.2. Effects of Sleep Deprivation on Cognitive Performance in Persons at Risk for Hypertension

**Figure 1:**
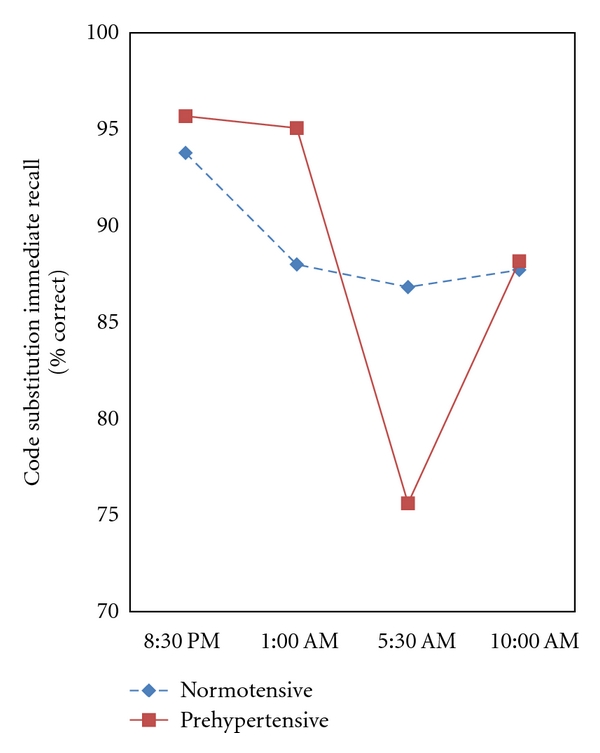
The effect of sleep deprivation on immediate code substitution recall (% correct) in persons classified by JNC-7 criteria of prehypertensive or normotensive. The time by Risk Group interaction was significant [multivariate *F*(3, 41) = 4.073, *P* = .013, *η* ^2^ = .230]. Fisher's LSD indicates a significant drop in performance at 5:30 AM, in prehypertensives (*P* = .03).

**Figure 2:**
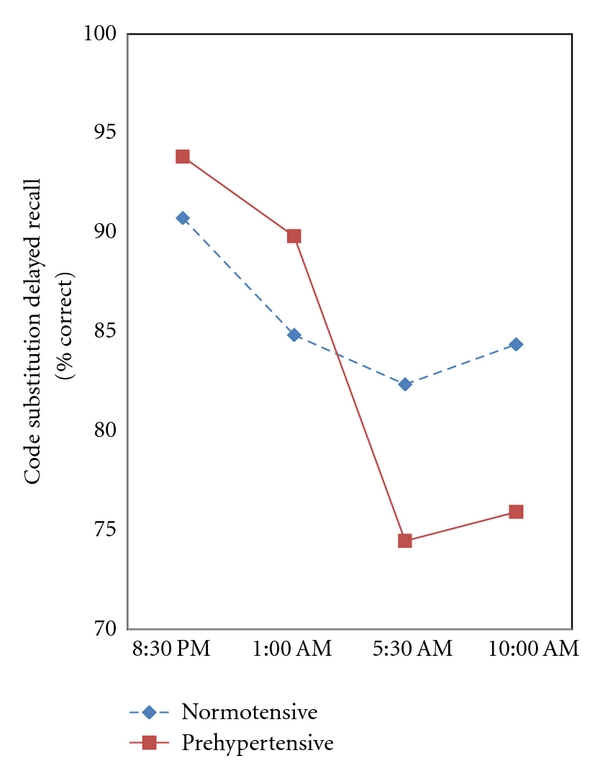
The effect of sleep deprivation on delayed code substitution recall (% correct) in persons classified by JNC-7 criteria of prehypertensive or normotensive. The time by Risk Group interaction was significant [multivariate *F*(3, 41) = 4.359, *P* = .009, *η* ^2^ = .242]. Fisher's LSD indicates a significant drop in performance at 5:30 AM (*P* = .009) and 10:00 AM (*P* = .017) in prehypertensives.

Repeated measures ANOVAs show significantly greater declines in cognitive performance of prehypertensives versus normotensives over the period of sleep deprivation. For example, Figure 1 shows the significant Risk Group × Time interaction for immediate code substitution memory performance [*F*(3, 41) = 4.073, *P* = .013, *η* ^2^ = .230]. Unlike normotensives, prehypertensives showed a large decline in memory performance at the 5:00 AM test (*P* = .03), but their performance accuracy recovered by the subsequent 10:00 AM test.  Figure 2 shows the significant Risk Group × Time interaction for delayed code substitution memory performance [*F*(3, 41) = 4.359, *P* = .009, *η* ^2^ = .242]. Relative to normotensives, prehypertensives showed a large decline in memory at the 5:00 AM test (*P* = .009) that remained through the subsequent 10:00 AM test (*P* = .017). A similar Risk Group × Time interaction was observed for category recall [*F*(3, 45) = 3.288, *P* = .029, *η* ^2^ = .180]. In this case, the performance decline in prehypertensives did not emerge until the 10:00 AM test (*P* = .046). For the Sternberg memory task, a significant Risk Group × Time × Sex interaction achieved statistical significance [*F*(3, 41) = 3.248, *P* = .031, *η* ^2^ = .192], indicating a decline in performance at the 10:00 AM testing in prehypertensive women.

**Figure 3:**
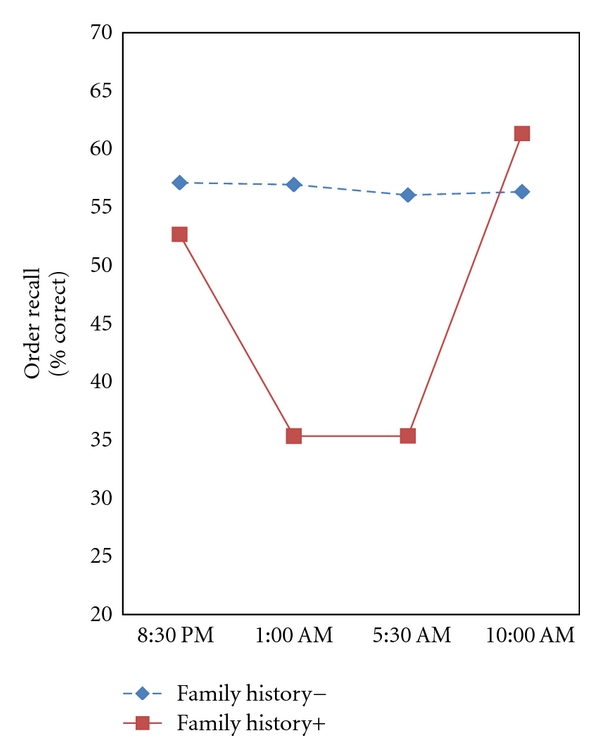
The effect of sleep deprivation on the order recall task (% correct) in persons classified as positive or negative family history of hypertension. The time by Risk Group interaction was significant [multivariate *F*(3, 21) = 4.061, *P* = .020, *η* ^2^ = .367].

Using family history as the risk variable, a significant Risk Group × Time interaction was observed for order recall [*F*(3, 21) = 4.061, *P* = .020, *η* ^2^ = .367]. In this case, persons with a positive family history showed a decline in order recall performance at 1:00 AM and 5:00 AM, with a relative recovery by the 10:00 AM testing. The effect of family history of hypertension over time is shown in Figure 3. No other Risk Group × Time interactions achieved statistical significance.

### 3.3. Relationship between Sleep-Deprivation-Induced Changes in Cognitive Function and Blood Pressures across the Night

Throughout the night of sleep deprivation, blood pressure was determined prior to each cognitive testing session. Therefore, we also examined the correlations between sleep-deprivation-induced changes in blood pressure and cognitive function across the night. While most correlations were not statistically significant, we did observe a pattern of significant positive correlations (2-tailed probabilities) between blood pressure and corresponding cognitive function, especially at the 1:00 AM test session. For example, at the 1:00 AM test session, SBP was positively correlated with performance on simultaneous code substitution (*r*(49) = .526, *P* < .001), Sternberg memory task (*r*(49) = .335, *P* = .023) and the continuous performance task (*r*(49) = .435, *P* = .003). Similarly, DBP was significantly correlated with performance on simultaneous code substitution (*r*(49) = .394, *P* = .007) and the Sternberg memory task (*r*(49) = .412, *P* = .004) at the 1:00 AM test session.

## 4. Discussion

Sleep deprivation has been shown to impair functioning of the distributed thalamoprefrontal cortical networks subserving attention and higher order cognitive processes [16, 17]. Consistent with these and other studies of sleep deprivation, measures of higher cognitive performance declined over time across all participants, regardless of risk status [22, 33]. However, our results show significantly greater cognitive declines in otherwise healthy young adults at risk for hypertension. No consistent differences between high- and low-risk groups in cognitive function were seen at the earlier times of testing, but the cognitive decline in high risk groups emerged most consistently at the 5:30 AM and 10:00 AM tests, after significant sleep deprivation. This suggests that subtle cognitive differences between high- and low-risk groups became apparent only after significant sleep deprivation had occurred. Persons at risk did not show exaggerated cognitive declines in relatively simple tasks, even after significant sleep deprivation. However, high-risk groups showed larger performance decrements across time in tasks requiring significant sustained attention and working memory. For example, there were no interactions of risk groups across time on code substitution learning with simultaneous display, but significant declines were seen for code substitution in both immediate and delayed recall tasks. Interestingly, memory performance of prehypertensives degraded on the more difficult delayed recall task by the 5:30 AM testing and remained low through the final 10:30 AM test session. In contrast, performance on the easier immediate recall task declined in prehypertensives at the 5:30 AM tests, but showed recovery at the 10:00 AM testing. This pattern likely reflects the partial recovery of alertness and cortical arousal associated with the rise in circadian rhythms entrained by sunrise in the light/dark cycle [34]. Prehypertensives also showed greater declines in performance over time in the category recall task. In addition, the decline in Sternberg recall performance in prehypertensives was confined primarily to women. Because of the small sample size in this group, this result should be interpreted with caution until confirmatory evidence is available.

Interestingly, we also observed a decline in order recall performance in persons with a positive family history of hypertension. Because there were no initial differences in resting blood pressure between positive and negative family history groups, this finding suggests that even mild elevations in resting pressure are not necessary for expression of the association between risk for hypertension and cognitive performance decline. This suggests that CNS changes may occur before significant blood pressure dysregulation.

### 4.1. Sleep Deprivation, Blood Pressure Control, and Cognition

Sleep deprivation and/or disruption can influence the sympathetic nervous system and blood pressure acutely [21] and via chronic sleep loss [34, 35]. However in the present study, scores on the Pittsburgh Sleep Quality Index global scale showed no group differences in chronic sleep quality, regardless of risk categorization (all *P*s >  .1). Thus, it is unlikely that the differential effects of acute sleep deprivation on these risk groups resulted from lower chronic sleep quality in high-risk groups.

Interestingly, sleep-deprivation-induced changes in acute blood pressure and cognition across the night showed a trend for positive correlations, especially at the 1:00 AM testing. This suggests that individuals with the worst cognitive performance at this time of night also showed signs of decreased arousal as indexed by lower acute blood pressure at the time of testing. This may reflect the relationship between cortical and autonomic arousal and is consistent with reports of decreases in blood pressure and attentiveness with increasing fatigue [36].

Overall, the cognitive decline in persons with mild elevations in resting blood pressure (JNC-7 prehypertensive) is consistent with results of Ditto et al. [14], Thyrum et al. [15], and others [13]. To our knowledge, this is the first study to report cognitive declines in persons with a positive family history of hypertension, without concomitant elevations in chronic resting blood pressure. The effect of family history was observed only for the order recall task, and additional research is needed to confirm the role of family history without associated elevations in resting blood pressure. Nevertheless, the present results clearly indicate that the systemic stress and fatigue of sleep deprivation exposes occult cognitive/CNS changes in otherwise healthy young adults at risk for hypertension development later in life.

### 4.2. Brain Function and Blood Pressure Control Mechanisms

Changes in cognitive function in younger persons without a history of significant and sustained high blood pressure may provide insight into the early etiological mechanisms in the developmental pathophysiology of hypertension. It is possible that even modest, subclinical resting blood pressure elevations could directly engender subtle CNS functional damage [14]. However, our finding of cognitive decline in a group of young adults with familial hypertension raises the possibility that functional CNS changes may be occurring in persons at risk before the development of mild elevations in chronic resting blood pressure. Nevertheless, persons with familial hypertension have increased blood pressure [18] and HPA reactivity to psychological stress [20], so it is possible that these periodic stress-associated blood pressure elevations may influence CNS function, even in the absence of modest elevations in chronic resting blood pressure. Additionally, stress can impair working memory in association with systemic cortisol release [37]. The precise role of glucocorticoids and other stress hormones in the observed cognitive declines in groups at risk remains to be determined. Additional research is needed to further explore the potential effect of acute, stress-induced elevations in blood pressure and stress hormones on brain function.

Notwithstanding the above, there are other possible links between blood pressure and CNS function in otherwise healthy young adults at risk. For example, CNS changes could be involved intimately with the progressive blood pressure dysregulation that eventuates in frank essential hypertension later in life. Several pathways could influence this process. Firstly, changes in CNS function and blood pressure control could be related indirectly with each other, but correlated with a heretofore unknown, underlying mechanism affecting both through distinct, but noninteractive pathways. Secondly, and even more intriguingly, CNS changes could contribute directly to dysfunction of autonomic and neuroendocrine systems involved in regulation of blood pressure. Under the latter scenario, changes in higher CNS function could cascade distally via central control of sympathoadrenomedullary, hypothalamic pituitary adrenocortical (HPA), and perhaps other neuroendocrine axes [38, 39] to provoke the blood pressure dysregulation observed in the early stages of hypertension development.

In support of this notion, an accumulating body of evidence points to a broad spectrum of subtle CNS changes in otherwise healthy persons with either mildly elevated blood pressure or normal resting blood pressure with other risk factors such as a positive history of hypertension. For example, prior work in our lab has shown abnormalities in opioidergic inhibition of both the HPA and the sympathoadrenomedullary axes in persons at risk for hypertension [19], suggesting alteration in brain mechanisms, either at or proximal to the paraventricular hypothalamus. Moreover, a large literature shows that hypertensive humans and animals as well as persons at risk for hypertension show reduced responsivity to pain [40, 41]. Recent findings suggest that changes in affective pain sensitivity may reflect a more generalized emotional dampening [42, 43] and may result in impaired perception of affective environmental cues [44]. This raises the possibility that subtle changes in brain function and performance may actually contribute to the autonomic dysregulation of blood pressure in persons at risk. For example, it is likely that modest changes in appraisal of threatening stimuli and/or memory function could directly contribute to increased psychological and/or psychosocial distress, with its consequent autonomic disturbance and blood pressure dysregulation. A dampening in threat appraisal could reduce motivation and directly contribute to reduced performance on complex tasks, including the cognitive learning and memory tasks.

The notion of CNS changes preceding blood pressure dysregulation is consistent with a recent report of reduced cry response to painful vitamin K injections in newborn infants with hypertensive grandparents [45]. Therefore, genetic and/or maternal stress hormone-based changes in fetal nervous system development [46, 47] could provoke subtle alterations in brain function, blood pressure control, and programming for adult disease. Thus, the notion of preexisting changes in CNS function in persons at risk for hypertension provides explanation for a large body of data and suggests a novel new possibility for CNS origins of blood pressure dysregulation in the developmental etiology of essential hypertension.

### 4.3. Methodological Limitations

Several limitations should be considered in interpretation of the present results. First, the 28-hour sleep deprivation methodology requires significant costs, as well as intensive time burden on participants. Thus, modest samples sizes are typical of studies where sleep deprivation is experimentally manipulated. Although a larger sample size would have been preferable, the present study nevertheless produced several significant results in line with hypothesized outcomes. This suggests that the current sample size has sufficient statistical power to expose reliable links between brain function and hypertension risk. Moreover, the experimental manipulation of sleep deprivation in the current study avoids the many potential covariates that confound correlational epidemiological studies of chronic sleep loss. Nevertheless, risk group differences in aerobic fitness or other related variables may have affected these results, despite similarities in body weight and BMI. Interestingly, only two subjects were classified as both prehypertensive and positive family history. This finding could reflect the relatively young age of the present sample. For example, the categorization by family history in our sample could favor a later developing blood pressure rise. An older study sample might show more persons with a family history of hypertension in the prehypertensive blood pressure range. Regardless of methodological limitations, the present results suggest that healthy young adults at risk for hypertension show decreased memory performance during a single night of sleep deprivation.

### 4.4. Conclusions

The overall findings of this study indicate that young adults at risk for hypertension show decreases in higher cognitive function during sleep deprivation. The sleep deprivation-induced cognitive declines in persons at enhanced risk are significantly greater than those seen in their low-risk counterparts. These risk-related cognitive declines are seen primarily in tasks that require sustained attention and working memory and emerge only after significant sleep deprivation. These results suggest that some cognitive effects in hypertensive patients may reflect CNS changes that occur prior to development of significant and sustained high blood pressure. Taken together with other findings, these results suggest that CNS changes may parallel, precede, or even contribute to blood pressure dysregulation in the early stages of the development of hypertension. Better understanding of the potential CNS origins of essential hypertension could lead to new strategies for treatment and prevention of this costly and widespread disease.

## Figures and Tables

**Table 1:** Means (± standard errors) for systolic and diastolic blood pressure and cognitive performance accuracy across a night of sleep deprivation with sustained cognitive work. All cognitive performance data are in percent correct.

	8:30 PM	1:00 AM	5:30 AM	10:00 AM
Systolic blood pressure (mmHg)	113.0 (1.64)	112.6 (1.78)	111.5 (1.72)	112.0 (1.57)
Diastolic blood pressure (mmHg)	66.3 (1.13)	67.2 (1.03)	66.6 (1.22)	66.5 (1.00)
Category recall	74.2 (2.05)	75.1 (2.42)	69.9 (2.58)	72.2 (2.41)
Code substitution learning	96.6 (.40)	94.9 (.66)	91.4 (.87)	92.7 (.90)
Code substitution immediate recall	93.3 (1.16)	88.5 (2.18)	83.2 (2.73)	86.9 (1.92)
Code substitution delayed recall	91.5 (1.53)	85.0 (2.37)	80.2 (2.18)	81.0 (2.31)
Sternberg memory recall	96.7 (.50)	91.6 (1.20)	90.1 (1.87)	86.7 (1.99)
Continuous performance test	90.2 (1.15)	83.9 (2.35)	76.6 (2.32)	75.3 (2.56)
